# The cytoskeletal regulator Coronin-1A plays a multidirectional role in glioblastoma stemness

**DOI:** 10.1038/s41598-026-55120-9

**Published:** 2026-05-28

**Authors:** TingTing Zhang, Ichiyo Shibahara, Takuichiro Hide, Toshihiro Kumabe, Shun-Ichiro Ogura, Tetsuya Taga, Kouichi Tabu

**Affiliations:** 1 Department of Stem Cell Regulation, Division of Visionary Life Science, Medical Research Laboratory, Institute of Integrated Research, Institute of Science Tokyo, Tokyo, 1138510 Japan; 2 Department of Neurosurgery, Kitasato University School of Medicine, Sagamihara, Kanagawa Japan; 3 School of Life Science and Technology, Institute of Science Tokyo, Yokohama, Japan; 4 Present Address: Department of Robotic Science, Division of Biological Data Science, Medical Research Laboratory, Institute of Integrated Research, Institute of Science Tokyo, Tokyo, Japan

**Keywords:** Cancer, Cell biology, Molecular biology, Oncology

## Abstract

Glioblastoma (GBM) recurrence is driven by therapy-resistant cells that escape surgical detection and withstand subsequent chemoradiotherapy. However, the molecular basis connecting invasion, metabolic detectability, and treatment resistance remains elusive. Here, we identify Coronin-1A (Coro1A), a cytoplasmic actin-associated regulator, as a multidirectional modulator of cancer stemness in GBM. Across six patient-derived GBM lines, Coro1A mRNA expression strongly correlated with both cell motility and the proportion of 5-aminolevulinic acid (5-ALA)–negative cells, and high Coro1A levels predicted significantly poorer prognosis in recurrent GBM cases. To assess its function, Coro1A-knockdown clones were generated from a recurrent GBM–derived culture (PDM123). Silencing Coro1A significantly reduced migration and modestly decreased proliferation, with the extent of motility reduction correlating with residual Coro1A expression. Importantly, knockdown enhanced intracellular accumulation of protoporphyrin IX (PpIX) under 5-ALA treatment, even in highly motile cells, indicating that Coro1A simultaneously governs invasiveness and intraoperative fluorescence detectability. Furthermore, Coro1A depletion increased sensitivity to temozolomide (TMZ) and X-ray irradiation, revealing its critical contribution to therapy resistance. Collectively, these findings establish Coro1A as a central cytoskeletal regulator that unifies invasion, diagnostic escape, and therapeutic resilience—highlighting its potential as a curative target capable of dismantling the multifaceted resistance of GBM.

**Supplementary Information:**

The online version contains supplementary material available at 10.1038/s41598-026-55120-9.

## Introduction

Glioblastoma (GBM) remains the most malignant primary brain tumor, with median survival barely exceeding 15 months despite maximal multimodal therapy^1–3^. Recurrence is almost inevitable, even after aggressive resection and adjuvant chemoradiotherapy, reflecting the persistence of a therapy-resistant subpopulation that survives standard treatments^4–7^. Intraoperative visualization using 5-aminolevulinic acid (5-ALA) has improved the precision of tumor removal by highlighting malignant tissues through the accumulation of protoporphyrin IX (PpIX)^8–13^. Nevertheless, cells that remain undetectable by this technique frequently drive relapse, underscoring the existence of metabolically evasive and treatment-resistant tumor cells that escape both surgical and pharmacological control.

These residual cells are increasingly recognized to possess cancer-stem-like properties—capable of self-renewal, adaptive survival, and reconstitution of tumor heterogeneity^14–17^. Such cancer stem cells (CSCs) display not only enhanced invasive potential but also remarkable resilience to cytotoxic stress^15,17^. We recently demonstrated that adaptive subsets of CSCs can remodel the immune niche and coordinate tumor resilience through altruistic self-restraint, redefining the functional identity of stemness in GBM^18–20^. However, the molecular programs that connect physical invasion, metabolic detectability, and therapeutic resistance at the single-cell level remain largely unknown.

Among the diverse mechanisms conferring aggressiveness, cytoskeletal remodeling is central to tumor invasion and dissemination^21–23^. Actin-associated regulators orchestrate cellular motility, adhesion, and morphological plasticity—processes essential for infiltrative growth of GBM^23–25^. Yet, while the importance of cytoskeletal dynamics in invasion has been well documented, whether cytoskeletal regulators also contribute to metabolic and therapeutic adaptability, key features of CSC-driven recurrence, is still poorly understood^21,24,26–28^. Bridging these domains could reveal pivotal nodes capable of synchronizing multiple malignant phenotypes.

Coronin-1A (Coro1A) is a cytoplasmic actin-binding protein that modulates actin turnover and signal transduction in immune cells, governing processes such as chemotaxis and phagocytosis^29–32^. Beyond the immune system, Coro1A expression has been sporadically noted in several cancers, but its functional significance remains unclear. Given its dual roles in cytoskeletal regulation and intracellular signaling, we hypothesized that Coro1A might serve as a convergence point linking motility, metabolic detectability, and therapy resistance—three hallmarks of malignant persistence in GBM.

In this study, we analyzed Coro1A expression across six patient-derived GBM lines representing diverse clinical backgrounds. We found that Coro1A mRNA levels were strongly correlated with both cell motility and the fraction of 5-ALA–negative cells, and that high Coro1A expression predicted poor prognosis in recurrent GBM patients. Functional assays using Coro1A-knockdown clones derived from a recurrent GBM culture demonstrated that Coro1A silencing significantly reduced migration, enhanced PpIX accumulation under 5-ALA exposure, and increased sensitivity to temozolomide (TMZ) and X-ray irradiation. These results indicate that Coro1A coordinates invasion, diagnostic detectability, and therapy resistance within a single regulatory framework.

Collectively, our findings identify Coronin-1A as a multidirectional regulator of glioblastoma stemness. By functionally integrating cytoskeletal behavior, metabolic escape, and treatment resilience, Coro1A embodies a unifying principle of GBM persistence and highlights an attractive molecular target with curative potential against this intractable disease^33^.

## Materials and methods

### Cell lines and culture

Patient-derived glioblastoma models used in this study are summarized in Table 1. Six patient-derived GBM cell lines (KBT#12137, KBT#10135, and KBT#10170) were established from surgical specimens obtained with written informed consent at Kumamoto University Hospital (Kumamoto, Japan) under institutional approval. Recurrent patient-derived GBM lines PDM19, PDM22, and PDM123 were obtained from the American Type Culture Collection via Summit Pharmaceuticals International (Tokyo, Japan), which supplies human-derived cell lines collected under appropriate ethical approval and patient consent. Clinical metadata for these models were obtained from ATCC model records and the Human Cancer Models Initiative (HCMI) database. Cells were maintained in Dulbecco’s modified Eagle’s medium/F-12 (DMEM/F-12; Gibco, Thermo Fisher Scientific, Waltham, MA, USA) supplemented with B27 (1:50; Invitrogen, Thermo Fisher Scientific), basic fibroblast growth factor (bFGF, 20 ng/ml; PeproTech, Cranbury, NJ, USA), and epidermal growth factor (EGF, 20 ng/ml; PeproTech) under non-adherent sphere culture, or in 10% fetal bovine serum (FBS) adherent conditions when indicated. All experiments were performed using mycoplasma-free cultures maintained at 37 °C in a humidified 5% CO₂ incubator.

**Table 1:** Clinical information of patient-derived glioblastoma models used in this study

Cell line	Diagnosis	Histological subtype	WHO grade	Clinical status	Age	Sex	Source
KBT#12137	Glioblastoma	Not otherwise specified	IV	Primary	71	Male	Kumamoto University Hospital
KBT#10135	Giant cell glioblastoma	Not otherwise specified	IV	Primary	47	Female	Kumamoto University Hospital
KBT#10170	Glioblastoma	Not otherwise specified	IV	Primary	55	Male	Kumamoto University Hospital
PDM-19	Glioblastoma	Not otherwise specified	IV	Recurrent	56	Female	ATCC
PDM-22	Glioblastoma	Not otherwise specified	IV	Recurrent	58	Male	ATCC
PDM-123	Glioblastoma	Not otherwise specified	IV	Recurrent	52	Male	ATCC

Clinical metadata for KBT models were obtained from Kumamoto University Hospital. Clinical metadata for PDM models were obtained from ATCC model records and the Human Cancer Models Initiative (HCMI) database.

#### Transcriptome analysis

For transcriptome profiling, genome-wide cDNA microarray analysis was outsourced to the Chemicals Evaluation and Research Institute (CERI, Tokyo, Japan) and performed using the Agilent Human Whole Genome Oligo Microarray platform (4 × 44 K, G4112F) according to the manufacturer’s protocol. Total RNA was extracted using the RNeasy Mini Kit (Qiagen, Hilden, Germany), and RNA integrity was verified (RIN > 8.0) using the Bioanalyzer 2100. Fluorescently labeled cRNA was synthesized, hybridized onto the arrays, and scanned with the Agilent Microarray Scanner. Raw data were processed using Agilent Feature Extraction software (v12.1.1.1; Agilent Technologies) and normalized by the quantile method. Normalized signal intensities were log₂-transformed and used for subsequent analyses. Genes belonging to the Gene Ontology Biological Process (GOBP) term “locomotion” (GO:0040011) were retrieved from the Molecular Signatures Database (MSigDB; Broad Institute, Cambridge, MA, USA)^34^. Sixteen candidates (DAPK2, TIRAP, CD200, SORD, LAMA4, CORO1A, ZNF609, CFAP251, WNK1, MEIG1, BCR, CFAP44, INS, ADAM9, LYPLA2, and MAZ) were screened for prognostic relevance using the Cancer Genome Atlas (TCGA)-GBM dataset (HG-U133A) via GlioVis web application^35,36^. Kaplan–Meier survival analysis was performed using the GlioVis platform, which provides survival statistics based on TCGA datasets. Differences between high- and low-expression groups (median cutoff) were evaluated using log-rank and Wilcoxon tests. Survival statistics for all locomotion-related genes analyzed are summarized in Table 2. Normalized Coro1A expression values from the six GBM lines were then correlated with migration and 5-ALA fluorescence phenotypes using Pearson’s correlation coefficients.

**Table 2:** Survival associations of locomotion-related genes in GBM cohorts (GlioVis analysis)

Gene symbol	Gene name	Primary GBM Log-rank p	Primary GBM Wilcoxon p	Recurrent GBM Log-rank p	Recurrent GBM Wilcoxon p	Direction of association
DAPK2	Death associated protein kinase 2	0.4993	0.3477	0.9311	0.9583	Not significant
TIRAP	TIR domain containing adaptor protein	Not available	Not available	Not available	Not available	Not evaluated
CD200	CD200 molecule	0.2725	0.5298	0.562	0.5311	Not significant
SORD	Sorbitol dehydrogenase	0.011	0.0011	0.8176	0.2912	Low expression associated with worse survival (Primary)
LAMA4	Laminin subunit alpha 4	0.0794	0.0736	0.7155	0.835	Not significant
CORO1A	Coronin 1A	0.195	0.5008	4.00E-04	9.00E-04	High expression associated with worse survival (Recurrent)
ZNF609	Ainc finger protein 609	0.9412	0.7582	0.2234	0.0382	Low expression associated with worse survival (Recurrent)
CFAP251	Cilia and flagella associated protein 251	Not available	Not available	Not available	Not available	Not evaluated
WNK1	WNK lysine deficient protein kinase 1	0.0721	0.0986	0.6463	0.5311	Not significant
MEIG1	Meiosis/spermiogenesis associated 1	Not available	Not available	Not available	Not available	Not evaluated
BCR	BCR activator of RhoGEF and GTPase	0.1679	0.0991	0.0023	0.0055	Low expression associated with worse survival (Recurrent)
CFAP44	Cilia and flagella associated protein 44	Not available	Not available	Not available	Not available	Not evaluated
INS	Insulin	0.3004	0.3736	0.6204	0.7938	Not significant
ADAM9	ADAM metallopeptidase domain 9	0.1232	0.2036	0.777	0.7545	Not significant
LYPLA2	Lysophospholipase 2	0.5716	0.5029	0.116	0.0374	Low expression associated with worse survival (Recurrent)
MAZ	MYC associated zinc finger protein	0.1722	0.4534	0.0357	0.0408	Low expression associated with worse survival (Recurrent)

#### Establishment of Coro1A-knockdown cells

The pGFP-C-shLenti vector encoding shRNA against human Coro1A was purchased from Origene Technologies (Rockville, MD, USA; Cat. No. TR30023). The effective shRNA sequences were: #A, 5′-GACACCAACATCGTCTACCTCTGTGGCAA-3′; and

#B, 5′-AAGTCGGACCTGTTCCAGGAGGACCTGTA-3′. A scrambled shRNA sequence (5′-GCACTACCAGAGCTAACTCAGATAGTACT-3′; non-targeting control, Origene, USA) was used as a negative control. Lentiviral particles were produced in HEK293T cells using the packaging plasmids pMDLg/pRRE, pCMV-VSV-G, and pRSV-Rev (Addgene, Watertown, MA, USA). To enhance yield, 10 µM forskolin (Tokyo Chemical Industry Co., Ltd, Japan) was added 24 h after co-transfection. PDM123 cells were transduced with viral supernatants in the presence of 10 µg/ml polybrene, followed by puromycin selection (2 µg/ml). Knockdown efficiency was verified by PCR using KOD One PCR Master Mix (TOYOBO, Japan). Primer sequences were as follows: Coro1A forward, 5′-CACTGTCGTAGCTGAGAAGGACCGTC-3′; reverse, 5′-GTTGCTATCCAGGCTGTGCTATC-3′; Actb forward, 5′-AGCGGGAGCACCATGTTCTCACCTTTAC-3′; reverse, 5′-CTCGTAGCTCTTCTCCAGGGAG-3′. To validate Coro1A depletion at the protein level, total protein was extracted from sphere-cultured parental, scramble control, and Coro1A-knockdown PDM123 cells using lysis buffer containing 10 mM Tris-HCl (pH 7.5), 150 mM NaCl, 5 mM EDTA, 10% glycerol, and 1% Triton X-100, supplemented with protease inhibitors (Complete, Roche; p-APMSF, FUJIFILM Wako Pure Chemical Corporation, Japan). Due to limited protein availability from parental sphere cultures, 5 μg of parental lysate and 15 μg of lysates from scramble control and Coro1A-knockdown cells were loaded per lane. Proteins were separated by SDS–PAGE using 5–20% precast gels (FUJIFILM Wako Pure Chemical Corporation, Japan) and transferred onto PVDF membranes (Amersham). Membranes were blocked with 5% skim milk (FUJIFILM Wako Pure Chemical Corporation, Japan) and incubated overnight at 4°C with primary antibodies against Coro1A (rabbit monoclonal, EPR19467-36, ab203698, Abcam; 1:500) and β-actin (mouse monoclonal, AC-15, A5441, Sigma-Aldrich; 1:1000). Membranes were then incubated with HRP-conjugated secondary antibodies, including goat anti-rabbit IgG (31460, Invitrogen) and goat anti-mouse IgG (31430, Invitrogen), and protein signals were visualized using a Fusion FX imaging system (Vilber).

#### Migration and proliferation assays

Cell motility was assessed using 24-well Transwell chambers with 8-µm pore polycarbonate membranes (Corning, #3422; Corning Inc., Corning, NY, USA). Single-cell suspensions (2 × 10^5^ cells/well) in serum-free DMEM/F-12 were seeded into the upper chamber, and complete sphere medium was placed in the lower chamber as a chemoattractant. After 72 h of incubation, cells that had migrated to the lower chamber and those remaining in the upper chamber were separately collected. Both fractions were subjected to 5-ALA treatment as described below to evaluate the relationship between migratory activity and PpIX accumulation. Migration assays were performed in triplicate using independent cultures. Cell proliferation was assessed using the Cell Counting Kit-8 (CCK-8; Dojindo Molecular Technologies, Kumamoto, Japan) after 72 h of culture. The absorbance was measured by analyzing the OD at 570 nm for each sample after 6 h and subtracting the background with OD at 630 nm, using a microplate reader (Bio-Rad, USA). All experiments were performed in triplicate.

#### 5-ALA treatment and PpIX fluorescence analysis

Cells were incubated with 1 mM 5-aminolevulinic acid (5-ALA) for 4 h at 37 °C under 5% CO₂. After incubation, PpIX fluorescence was analyzed by flow cytometry (FACS Aria II; BD Biosciences, San Jose, CA, USA). Fluorescence was excited with a 488 nm laser and detected using a 660/20 nm band-pass filter. Data were processed using FlowJo software (v7.6.5; TOMY Digital Biology, Tokyo, Japan)^37^. The proportion of PpIX-negative cells was calculated as mean ± SD from at least three independent experiments.

#### Chemoradiotherapy sensitivity assays

To evaluate therapeutic response, cells were exposed to TMZ or X-ray irradiation, reflecting the standard adjuvant modalities used in GBM treatment. TMZ (Tokyo Chemical Industry Co., Tokyo, Japan) was applied at 0, 10, 20, 50, and 100 µM , a concentration range commonly used in experimental studies of glioblastoma cells^38^. Although temozolomide readily penetrates the central nervous system, pharmacokinetic studies indicate that cerebrospinal fluid exposure is lower than plasma exposure^39^. Therefore, these in-vitro concentrations were not intended to directly reproduce intratumoral drug levels, but rather to evaluate comparative TMZ sensitivity under controlled experimental conditions. Cell viability was assessed after 72 h using the CCK-8 assay (Dojindo, Japan). For irradiation, cells were treated with 0, 15, or 60 Gy using an MX160-Labo X-ray irradiator (MediTEX, Chiba, Japan)^33^.

### Statistical analysis

All data are expressed as mean ± standard deviation (SD). Comparisons between two groups were performed using Student’s t-test, and correlations were analyzed using Pearson’s correlation coefficient. Survival differences in TCGA data were evaluated using Kaplan–Meier plots and log-rank tests. A p-value < 0.05 was considered statistically significant.

## Results

### Correlation of Coro1A expression with cell motility and 5-ALA fluorescence in GBM patient-derived cells

**Fig. 1:**
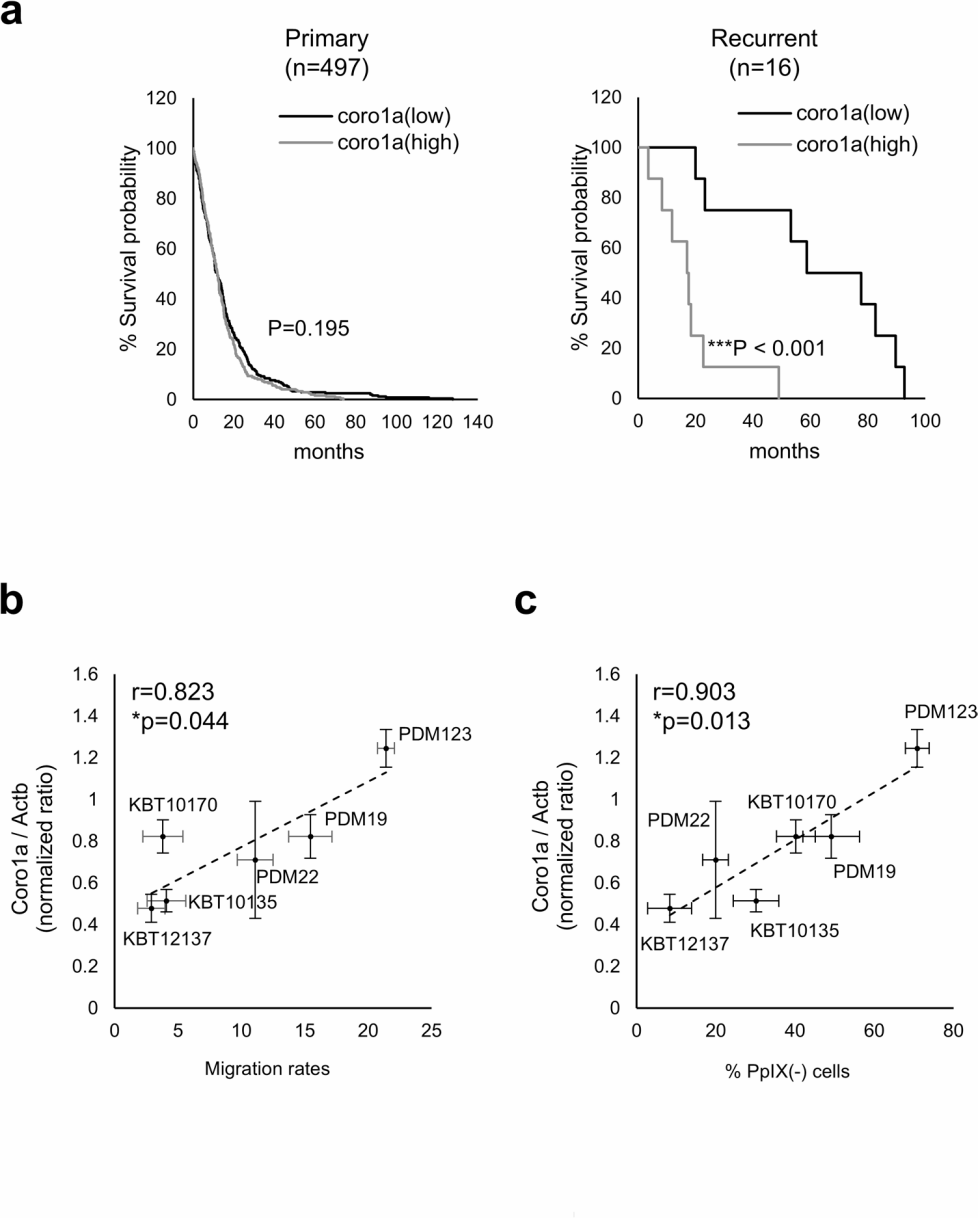
Identification of Coro1A as a locomotion-related gene associated with poor prognosis and invasive/fluorescence-escape phenotypes in GBM. (**a**) Overall-survival analysis of primary and recurrent GBM patients in the TCGA dataset comparing high and low Coro1A expression groups (log-rank ***p < 0.001 in Recurrent). Among 16 genes in the GOBP “locomotion” category, only CORO1A showed significance under the median-split condition in this dataset. (**b**) Correlation between Coro1A mRNA levels and cell-migration capacity (r = 0.823, *p < 0.05). (**c**) Correlation between Coro1A mRNA levels and the proportion of 5-ALA–negative cells (r = 0.903, *p < 0.05). Transcriptome-based Coro1A expression levels across six patient-derived GBM lines exhibiting distinct motility and 5-ALA–induced PpIX phenotypes. Each value represents the mean ± SD (*n* = 3 independent experiments).

To identify cytoskeletal regulators linked to GBM malignancy, we first screened genes categorized in the GOBP term “locomotion”. Among 16 candidate genes retrieved (DAPK2, TIRAP, CD200, SORD, LAMA4, CORO1A, ZNF609, CFAP251, WNK1, MEIG1, BCR, CFAP44, INS, ADAM9, LYPLA2, and MAZ), we examined their association with patient survival using the TCGA GBM dataset through the GlioVis platform. CORO1A emerged as the only gene whose high expression was significantly associated with poorer overall survival in recurrent GBM patients in TCGA dataset (Fig. 1a, log-rank *p* < 0.001). Although several genes showed statistically significant associations in certain analyses, these reflected the opposite trend (lower expression associated with poorer survival) or were not consistently observed across cohorts (Table 2). We therefore focused on Coronin-1A (Coro1A) for further analysis. To experimentally validate this association, we next examined Coro1A expression across six patient-derived GBM lines using their transcriptome datasets, from which quantitative mRNA expression levels were obtained. These lines exhibited distinct motility and 5-aminolevulinic acid (5-ALA)–induced PpIX accumulation phenotypes. Although Coro1A was not exclusively expressed in highly motile or PpIX-non-accumulating subpopulations, its expression levels showed a strong positive correlation with both migration capacity (r = 0.823, *p* < 0.05) and the proportion of 5-ALA–negative cells (r = 0.903, *p* < 0.05) (Fig. 1b,c). These data indicate that Coro1A upregulation, initially identified as a poor-prognosis marker in clinical datasets, is functionally associated with the invasive and fluorescence-escape phenotypes of GBM patient-derived cells.

#### Coro1A knockdown significantly reduces the migratory activity of recurrent GBM cells

**Fig. 2:**
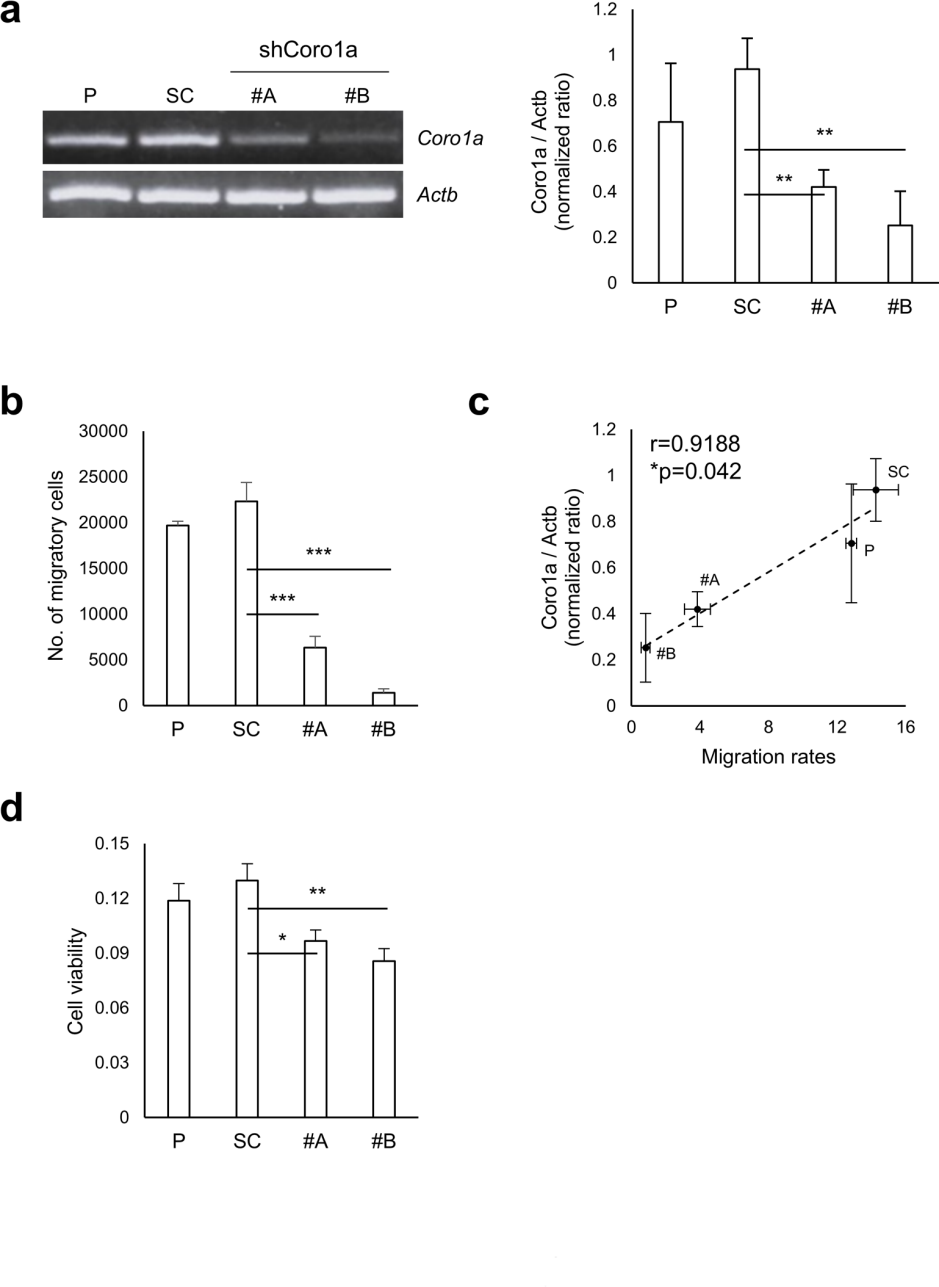
Coro1A knockdown significantly reduces the migratory activity of recurrent GBM cells. (**a**) qPCR verification of partial Coro1A knockdown in PDM123 recurrent GBM-derived clones #A and #B compared with scramble control (Sc). The left panel shows representative gel images of Coro1A and Actb amplicons, and the right panel presents densitometric quantification of relative Coro1A/Actb expression normalized to Sc levels (mean ± SD, **p < 0.01). (**b**) Transwell migration assays showing decreased motility in Coro1A-deficient cells. ***p < 0.001. (**c**) Correlation between residual Coro1A expression and relative migration rate (r = 0.9188, *p < 0.05). (**d**) Cell-proliferation assay showing a modest but significant reduction in growth of knockdown cells (*p < 0.05, **p <0.01). Statistical analysis was performed using Student’s t test; data are mean ± SD (*n* = 3 independent experiments).

To determine the functional role of Coro1A in GBM cells, we established two independent Coro1A-knockdown clones (#A and #B) from a recurrent GBM-derived line (PDM123). Quantitative PCR analyses confirmed a marked but incomplete reduction of Coro1A mRNA expression in both clones compared with scramble control cells, indicating partial knockdown (Fig. 2a, Supplementary Fig. S1). Western blot analysis further confirmed reduced Coro1A protein expression in knockdown clones compared with scramble control cells (Supplementary Fig. S2). Full-length uncropped blot images are provided in Supplementary Fig. S3. In Transwell migration assays, Coro1A knockdown significantly suppressed cell motility (Fig. 2b), with migration rates decreased by approximately 72% in clone #A and 94% in clone #B relative to Scramble controls (*p* < 0.001). Moreover, the extent of motility reduction correlated with residual Coro1A expression levels (r = 0.9188, *p* < 0.05), supporting a dose-dependent relationship between Coro1A expression and migratory capacity (Fig. 2c). Cell proliferation was also modestly but significantly reduced (*p* < 0.05 in #A, *p* < 0.01 in #B) (Fig. 2d), indicating that Coro1A contributes to both migration and growth potential of recurrent GBM cells.

#### Coro1A knockdown enhances 5-ALA–based PpIX accumulation

**Fig. 3:**
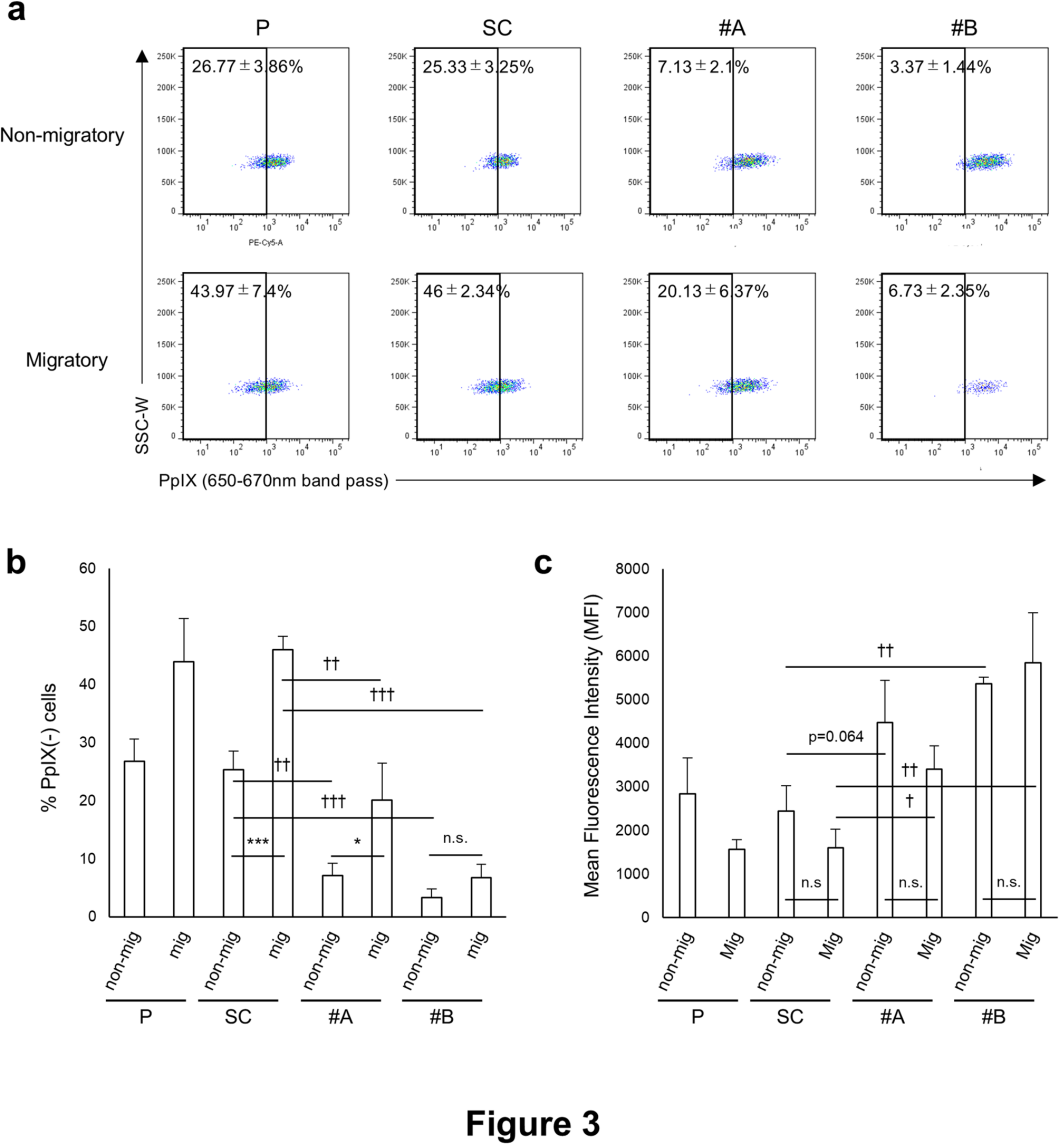
Coro1A knockdown enhances 5-ALA–based PpIX accumulation in recurrent GBM cells. (**a**) Representative fluorescence images of highly motile and less motile PDM123 cells showing intracellular PpIX accumulation after 5-ALA treatment in scramble (Sc) and Coro1A-knockdown clones. (**b**) Flow-cytometric quantification of PpIX non-accumulating cells. Coro1A knockdown significantly increased the proportion of PpIX-positive cells in both highly motile and less motile subpopulations. (**c**) Flow-cytometric quantification of PpIX mean fluorescence intensity (MFI) across the entire cell population in parental (P), scramble control (Sc), and Coro1A-knockdown cells (#A and #B). Values represent mean ± SD (*n* = 3 independent experiments). Statistical significance was determined using Student’s t-test. *p < 0.05 and ***p < 0.001 indicate comparisons between less motile and highly motile cells, whereas ^†^*p* < 0.05, ^††^*p* < 0.01, and ^†††^*p* < 0.001 indicate comparisons between scramble control and Coro1A-knockdown cells.

To determine whether Coro1A affects intraoperative fluorescence detectability, we analyzed 5-ALA–induced PpIX accumulation in control and Coro1A-knockdown cells. Remarkably, Coro1A silencing significantly reduced the proportion of PpIX-non-accumulating cells in both highly motile and less motile subpopulations (Fig. 3a,b). Consistently, flow-cytometric quantification of mean fluorescence intensity (MFI) demonstrated increased overall PpIX fluorescence in Coro1A-knockdown cells (Fig. 3c). These results indicate that Coro1A knockdown enhances intracellular PpIX accumulation, suggesting that Coro1A regulates not only cytoskeletal dynamics but also metabolic processes determining 5-ALA responsiveness, thereby functionally linking invasion and diagnostic escape within a single regulatory framework.

#### Coro1A depletion increases sensitivity to clinically relevant temozolomide and radiation doses

**Fig. 4:**
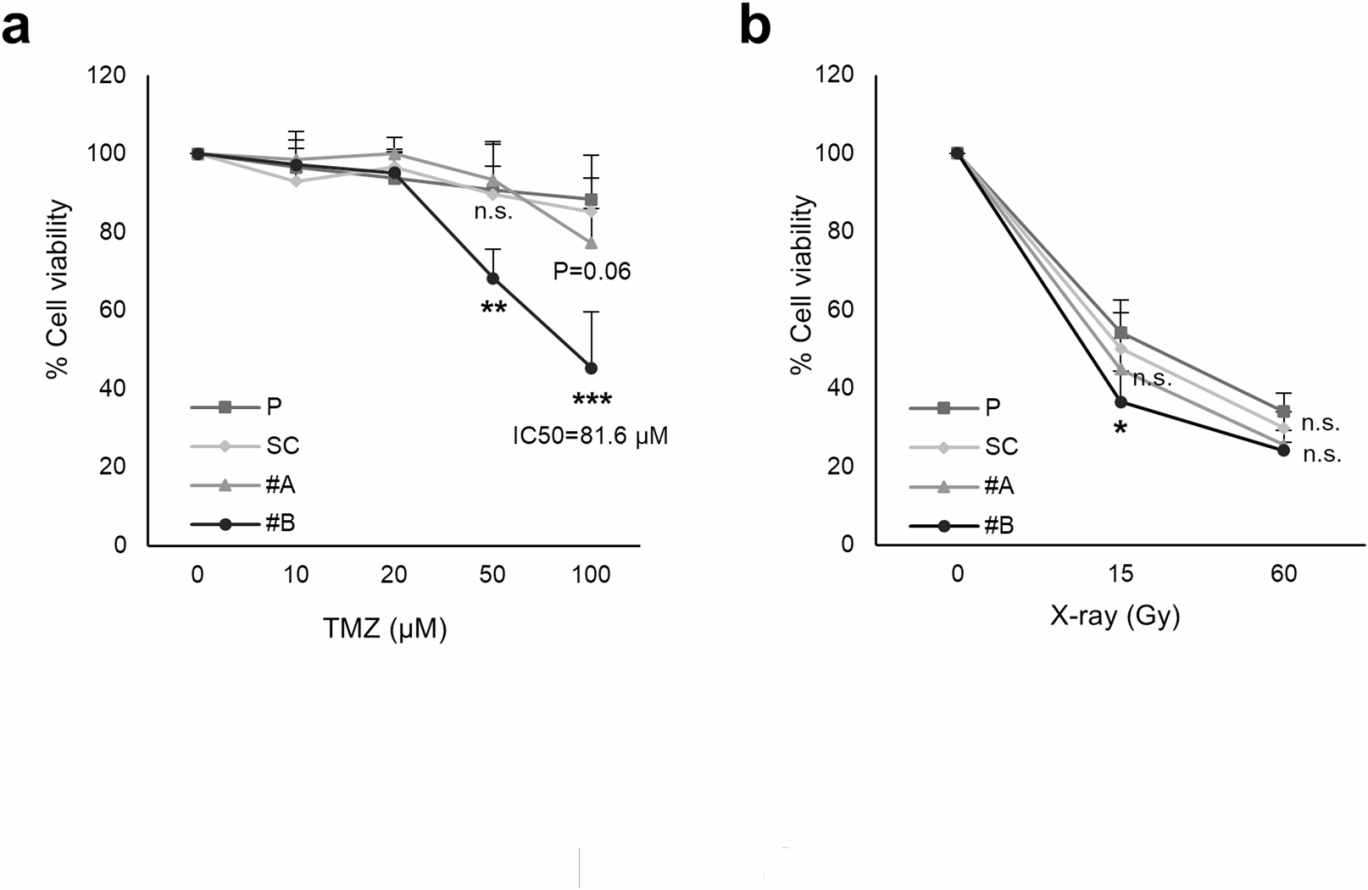
Coro1A depletion increases sensitivity to clinically relevant temozolomide and radiation doses. (**a**) Dose-dependent decrease in cell viability following TMZ treatment in parental PDM123 cells (P) and Coro1A-knocked-down (#A and #B) versus scramble control cells (Sc) (*p* < 0.01 at 50 μM and 100 μM). TMZ was applied at 0, 10, 20, 50, and 100 μM, reflecting plasma concentrations used in clinical protocols. (**b**) Decreased cell viability after X-ray irradiation in a Coro1A knocked-down clone #B compared with scramble control (Sc) at 15 Gy (*p < 0.05). Data are mean ± SD (*n* = 3 independent experiments); significance determined by Student’s t test.

To further explore the role of Coro1A in treatment resistance, we tested the response of recurrent GBM cells to TMZ and X-ray irradiation, which represent the standard therapeutic modalities used for glioma patients. Treatment conditions were designed to mirror clinically relevant ranges: TMZ was administered at 0, 10, 20, 50, and 100 μM, corresponding to plasma concentrations achievable during standard chemotherapy, and radiation was delivered at 0, 15, and 60 Gy, approximating cumulative doses used in clinical radiotherapy protocols. In these assays, Coro1A-knockdown clones exhibited a pronounced increase in treatment sensitivity compared with scramble controls. Cell viability following TMZ exposure decreased in a dose-dependent manner, with significant enhancement of cytotoxicity observed at 50 (*p* < 0.01) and 100 μM (*p* < 0.001) (Fig. 4a). Similarly, cell viability after irradiation was markedly reduced in Coro1A-deficient cells at both 15 Gy (*p* < 0.05 in #B) (Fig. 4b). These results demonstrate that Coro1A depletion restores susceptibility of recurrent GBM cells to standard postoperative chemoradiotherapy, suggesting the contribution of Coro1A to the multifaceted therapeutic resistance that underlies GBM recurrence.

## Discussion

This study identifies Coronin-1A (Coro1A) as a cytoplasmic regulator that integrates multiple malignant traits of GBM, including invasion, intraoperative fluorescence escape, and resistance to postoperative chemoradiotherapy. Because these properties collectively define the therapeutic obstacles that drive GBM recurrence, our findings highlight Coro1A as a potential curative target that could reinforce both surgical detectability and adjuvant efficacy. By demonstrating that Coro1A expression correlates with cell motility, 5-ALA–negative fraction, and poor patient prognosis, and that its knockdown restores sensitivity to TMZ and radiation, this study provides a unified explanation for the persistence of therapy-resistant GBM cells after multimodal treatment.

Members of the coronin family have been characterized as actin-binding proteins involved in cytoskeletal reorganization, vesicle trafficking, and immune cell motility^30,40–44^. Among them, Coro1A is best known for its role in leukocyte migration and phagosome formation, yet its function in cancer cells has remained largely unexplored^32,45^. Previous reports have suggested that coronins may influence metastatic potential or chemoresistance in certain solid tumors, but their involvement in glioma biology has been limited to transcriptomic correlations without functional validation^46–48^. The present study extends this knowledge by establishing a causal relationship between Coro1A expression and invasive as well as therapy-resistant behaviors in GBM. Notably, Coro1A was identified through a systematic screen of 16 locomotion-related genes and was the only candidate that showed a significant association with poor survival in recurrent GBM patients, underscoring its biological and clinical relevance. Although studies have found a opposite correlation between Coronin and tumor aggressiveness, this discrepancy may be due to isoform, tumor-type, or model differences^49^.

Mechanistically, Coro1A may exert its multidirectional control through actin-dependent processes that coordinate both cellular motility and metabolic adaptation^32,50,51^. Actin dynamics are increasingly recognized to influence mitochondrial distribution, endosomal trafficking, and intracellular signaling—all of which can affect 5-ALA metabolism and drug responsiveness. Coro1A, as a scaffolding molecule interacting with Rho GTPases and Arp2/3 complexes, could modulate these systems to fine-tune the balance between invasion and survival^51–53^. The observed enhancement of PpIX accumulation after Coro1A silencing suggests that Coro1A might regulate vesicular export or heme synthesis pathways that determine 5-ALA responsiveness. Similarly, the increased sensitivity to TMZ and irradiation implies that Coro1A supports stress-adaptive pathways, possibly by stabilizing cytoskeletal networks that confer resistance to DNA damage or oxidative stress. Further transcriptomic and proteomic profiling of Coro1A-interacting partners will be essential to clarify these mechanisms.

In the present study, we use the term “stemness” to describe malignant cellular states that enable tumor persistence after standard therapy. In GBM, recurrence is driven by cells that evade surgical detection through infiltrative growth and metabolic fluorescence escape and that survive postoperative chemoradiotherapy. We therefore operationally define GBM stemness as the capacity of tumor cells to resist surgical, metabolic, and therapeutic elimination, rather than relying solely on surrogate in-vitro assays such as sphere formation or stem-cell marker expression. From this perspective, the coordinated regulation of invasion, 5-ALA fluorescence escape, and chemoradiotherapy resistance observed in Coro1A-expressing cells represents a functional manifestation of GBM stemness in a clinically relevant context.

From a surgical viewpoint, 5-ALA–nonfluorescent invasive zones often harbor minor tumor subclones that are under-represented at primary resection but later expand under therapeutic selection, ultimately dominating at recurrence. This clinical observation aligns with our data showing that Coro1A links invasiveness with metabolic “fluorescence escape,” suggesting that targeting Coro1A could diminish precisely those nonfluorescent reservoirs that seed relapse. From a clinical standpoint, the present findings hold considerable translational potential. The use of TMZ concentrations and X-ray doses reflecting clinical regimens strengthens the validity of our results, indicating that Coro1A contributes to the therapeutic tolerance observed after standard postoperative treatment. However, the TMZ sensitization observed here should be interpreted within the context of an in-vitro dose-response assay, as the concentrations at which clear differences emerged (50–100 μM) do not necessarily correspond to clinically achieved intratumoral exposure. Nevertheless, the consistent shift in response under identical experimental conditions supports a role for Coro1A in modulating TMZ sensitivity. Importantly, partial knockdown of Coro1A sensitized recurrent GBM cells to both modalities, suggesting that Coro1A underlies the multifaceted therapeutic resistance driving GBM recurrence. Targeting Coro1A may therefore represent a dual-benefit strategy—enhancing intraoperative fluorescence detection while simultaneously improving the efficacy of adjuvant chemoradiotherapy. Such an approach could pave the way toward more complete eradication of residual GBM cells that escape current multimodal interventions.

This study has certain limitations. The analyses were primarily conducted using in-vitro models derived from recurrent GBM, and in-vivo validation will be required to confirm the therapeutic relevance of Coro1A targeting within the tumor microenvironment. In addition, functional validation experiments were performed using a single recurrent GBM model (PDM123), and future studies using additional patient-derived models will be important to further generalize these findings. Nonetheless, the consistent results obtained from patient-derived lines and clinical datasets strongly support the pathological importance of Coro1A. Future studies employing orthotopic xenograft or recurrence models will help clarify whether Coro1A suppression can indeed reduce tumor regrowth and improve survival outcomes. Meanwhile, Coro1A is expressed in a number of hematopoietic cells, including NK cells, macrophages, and lymphocytes, its ablation may have off-target effects on immune infiltration or angiogenesis, via Cre-loxP system may help establish the conditional knockout models. In addition, the development of pharmacological inhibitors or RNA-based therapeutics against Coro1A could provide new options for combinatorial therapy with standard chemoradiation.

In summary, this work establishes Coro1A as a multidirectional regulator of glioblastoma stemness that unifies cytoskeletal regulation, metabolic escape, and therapy resistance. By linking these malignant traits within a single molecular framework, Coro1A represents a promising therapeutic node capable of dismantling the multifaceted resistance of GBM. Targeting Coro1A may thus provide a novel avenue for achieving durable control or even cure of this intractable brain tumor.

## Supplementary Information

Below is the link to the electronic supplementary material.

Supplementary Material 1

Supplementary Material 2

Supplementary Material 3

## Data Availability

The data generated in the present study may be requested from the corresponding author.

## Abbreviations

5-ALA: 5-aminolevulinic acid
Actb: Beta-actin
bFGF: Basic fibroblast growth factor
CCK-8: Cell Counting Kit-8
Coro1A: Coronin-1A
CSC: Cancer stem cell
DMEM/F-12: Dulbecco’s modified Eagle’s medium/Nutrient Mixture F-12
EGF: Epidermal growth factor
FACS: Fluorescence-activated cell sorting
FBS: Fetal bovine serum
GBM: Glioblastoma
GOBP: Gene Ontology Biological Process
Gy: Gray
MSigDB: Molecular Signatures Database
PCR: Polymerase chain reaction
PpIX: Protoporphyrin IX
qPCR: Quantitative polymerase chain reaction
RIN: RNA integrity number
SD: Standard deviation
shRNA: Short hairpin RNA
TCGA: The Cancer Genome Atlas
TMZ: Temozolomide
